# The mitochondrial genome of the deep-sea limpet *Bathyacmaea nipponica* (Patellogastropoda: Pectinodontidae)

**DOI:** 10.1080/23802359.2019.1668732

**Published:** 2019-09-23

**Authors:** Jin Sun, Yaping Liu, Ting Xu, Yanjie Zhang, Chong Chen, Jian-Wen Qiu, Pei-Yuan Qian

**Affiliations:** a Department of Ocean Science, Division of Life Science and Hong Kong Branch of the Southern Marine Science and Engineering Guangdong Laboratory, The Hong Kong University of Science and Technology, Hong Kong, China;; b Department of Biology, Hong Kong Baptist University, Hong Kong, PR China;; c X-STAR, Japan Agency for Marine-Earth Science and Technology (JAMSTEC), Yokosuka, Japan

**Keywords:** *Bathyacmaea*, methane seep, South China Sea, Patellogastropoda

## Abstract

The deep-sea limpet *Bathyacmaea nipponica* is endemic to hydrothermal vents and hydrocarbon seeps in the Western Pacific. We report the complete mitochondrial genome of *B. nipponica*, which is 16,792 bp in length containing 13 protein-coding genes (PCGs), 22 *tRNA* genes, and two *rRNA* genes. Phylogenetic analysis using 13 PCGs shows that *B. nipponica* is within Patellogastropoda and is sister to a clade comprising *Cellana* and *Nacella*, among the taxa included.

Petallogastropoda, commonly referred to as true limpets, has long been considered as an early diverging group of Gastropoda (Bouchet et al. 2005), although recently much debated (Cunha and Giribet 2019). Molecular sequences within patellogastropods are limited compared to other gastropods, with an examination of the NCBI nr database in June 2019 revealed only seven complete mitogenomes. Among these, the mitogenome of *Lottia digitalis* exhibits accelerated evolutionary rate and thus could introduce long-branch attraction artifacts in phylogenetic analysis (Uribe et al. 2019).

To increase the diversity of molecular sequences within Petallogastropoda, we sequenced the mitogenome of the deep-sea patellogastropod *Bathyacmaea nipponica* Okutani et al. 1992 collected from a methane seep in the South China Sea (22° 06.948′N, 119° 17.116′E, depth 1138 m) in June 2013 by the manned deep-submergence vehicle *Jiaolong*. Upon recovery, limpets were immediately frozen in −20 °C and the samples were deposited at Hong Kong Baptist University with the sample accession number of Jiaolong_2013SCS_Bni. Genomic DNA was extracted using the CTAB method and sequenced by an Hiseq2500 (Illumina) sequencer (Xu et al. 2016; Sun et al. 2017). Approximately 6 Gb of reads were produced and the CLC workbench with the word size of ‘auto-size’ was used for assembly. One contig, deduced to be the mitogenome, was selected and a pair of primers was used to close the gap between the head and tail to complete the circular mitogenome. The CO1 sequence showed 99.34% similarity to *B. nipponica* collected from Off Hatsushima seep in Sagami Bay, Japan, the type locality of this species (Nakano and Ozawa 2007), indicating a wide distribution from Sagami Bay to the South China Sea.

The mitogenome was automatically annotated by the MITOS web server (Bernt et al. 2013) and sequentially manually corrected. The complete mitogenome of *B. nipponica* is 16,792 bp in length, encapsulating 13 protein-coding genes (PCGs), 22 *tRNA* genes, and two *rRNA* genes. The mitogenome was deposited in NCBI with the accession number of MF095859. The PCGs of ND1, ND5, ATP8, CO2, CO1, ND2, and CO3 genes start with ATG, while those of the rest start with ATA. All PCGs except ATP8 use the stop codon TAA, while ATP8 terminates with TAG. Comparisons with other gastropod mitogenomes show that the gene order of the 13 PCGs and 2 *rRNA* genes in the *B. nipponica* mitogenome is identical to that of the hypothetical ancestral gastropod (Uribe et al. 2019).

Phylogenetic analysis using the 13 PCGs following our published method (Sun et al. 2019) confirmed the placement of *B. nipponica* within Patellogastropoda (Figure 1). General phylogenetic positions of the six main gastropod lineages are consistent with previous studies using mitogenomes (Lee et al. 2019; Uribe et al. 2019) and Petellogastropoda is placed in a basal position among gastropods included. Recent phylogenomic evidences indicated a sister relationship between Patellogastropoda and Vetigastropoda (Cunha and Giribet 2019) which has not been recovered in mitogenome phylogenies and the true position of Patellogastropoda thus remains contested. The relative positions of the four patellogastropods included agrees with previous studies (Nakano and Ozawa 2007).

**Figure 1.**
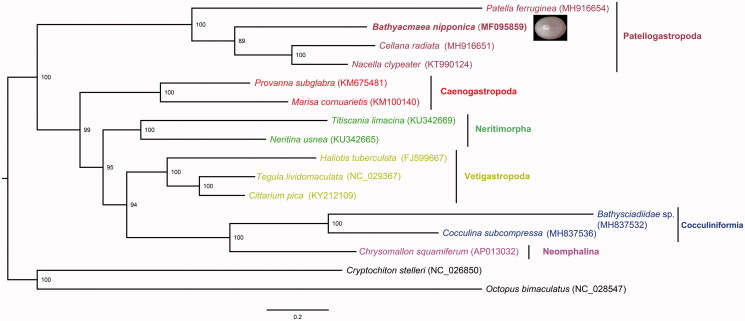
Phylogenetic reconstruction showing the relationship between *Bathyacmaea nipponica* and other gastropods. Phylogenetic analysis was performed using RAxML version 8.2.11 on 2552 distinct alignment residues from 13 protein coding genes. GTR + Γ was selected as the model for each partition. Nodal values indicate support in bootstrap values.
